# Refining intra-protein contact prediction by graph analysis

**DOI:** 10.1186/1471-2105-8-S5-S6

**Published:** 2007-05-24

**Authors:** Milana Frenkel-Morgenstern, Rachel Magid, Eran Eyal, Shmuel Pietrokovski

**Affiliations:** 1 Department of Molecular Genetics, Weizmann Institute of Science, Rehovot, 76100, Israel; 2 Department of Computational Biology, School of Medicine, University of Pittsburgh, Pittsburgh, PA 15213, USA

## Abstract

**Background:**

Accurate prediction of intra-protein residue contacts from sequence information will allow the prediction of protein structures. Basic predictions of such specific contacts can be further refined by jointly analyzing predicted contacts, and by adding information on the relative positions of contacts in the protein primary sequence.

**Results:**

We introduce a method for graph analysis refinement of intra-protein contacts, termed GARP. Our previously presented intra-contact prediction method by means of pair-to-pair substitution matrix (P2PConPred) was used to test the GARP method. In our approach, the top contact predictions obtained by a basic prediction method were used as edges to create a weighted graph. The edges were scored by a mutual clustering coefficient that identifies highly connected graph regions, and by the density of edges between the sequence regions of the edge nodes. A test set of 57 proteins with known structures was used to determine contacts. GARP improves the accuracy of the P2PConPred basic prediction method in whole proteins from 12% to 18%.

**Conclusion:**

Using a simple approach we increased the contact prediction accuracy of a basic method by 1.5 times. Our graph approach is simple to implement, can be used with various basic prediction methods, and can provide input for further downstream analyses.

## Background

The structure of proteins is determined by their amino acids sequence, with little or no other external information. Nevertheless, predictions of protein structure from their sequence information are still inaccurate. Protein structure is defined by the pattern and nature of the contacts between its amino acid residues. Contacts between nearby residues (typically 1–5 places apart) account for the protein secondary structure elements (*i.e*., alpha helices, beta strands and turns). Contacts between more distant residues determine the overall global protein structure. Accurately identifying a small part of such contacts is sufficient for predicting global protein structures [1]. Proteins evolve by mutations, gene duplications and functional selections. They can be organized in protein families of common origin and corresponding structure, which accumulated sequence changes. The patterns of these changes are a rich information source for identifying the structure of proteins in each protein family.

Co-variation, or correlated mutation, analysis is a powerful approach to identify pairs of co-evolving residues. Most frequently, the linkage between such residues is due to a direct contact between them [2]. An approach we recently developed identifies pairs of likely contacting residues by the similarity of their exchange patterns within a protein family with the a general pair-exchange matrix calculated from a very large amount of multiple sequence alignments and known structures [3]. Such approaches score the likelihood of protein residue pairs to contact each other. Some methods have been developed to refine these basic contact-prediction approaches by integrating the predictions of individual pairs. These methods add to the basic predictions other information, such as the relative positions of predicted contacts, the predicted secondary structure, and predicted solvent accessibility. All data is integrated by machine learning approaches, such as neural networks and HMMs [4-9]. The recent CASP competition for contact prediction demonstrated that methods making use of such peripheral information are usually better than the basic methods [10].

The PoCM method of Hamilton *et al *[6] is an advanced method, which uses neural networks to predict residue contacts in a protein. The main input to the neural network is a set of 25 measures of correlated mutation between all pairs of residues in two "windows" of size five centered on the given residues. It uses also predicted secondary structure of a protein and different residue classes such as nonpolar-hydrophobic, polar-hydrophilic, acidic or basic. Its accuracy is reported to achieve 30.7% for the top *L*/10 predictions (*L *being the length of the input protein).

We present here a new approach for refining basic intra-protein contact predictions based on graph analysis. Representing predicted contacts as graphs enables the identification of highly connected regions or local clusters in the graph. These correspond to contact networks that characterize protein structures [11]. We also seek for pairs of primary sequence regions, which are predicted to be joined by several contacts in windows. This procedure utilizes the modular nature of protein structure, where secondary structure elements (usually strands with strands and helices with helices) often interact with each other by several contacts. Finally, we focus our predictions on protein core regions. These regions include most of the contacts crucial for protein structure stability, and can be accurately predicted from sequence information alone [12].

## Results

To refine intra-protein contact predictions we first transformed basic contact prediction scores for a protein, which is represented by a multiple sequence alignment (MSA), into a graph (network). Each node in the graph corresponds to a protein residue (and its MSA column), and each edge corresponds to a predicted contact likelihood score between a pair of protein residues. For the pattern of edges (topology) to be informative, the graph should not be fully or regularly connected, the edges should be differentially weighted, or both. We chose to create sparse graphs from top scoring predictions (edges) and tested the approach with and without considering edge weights.

**Figure 1:**
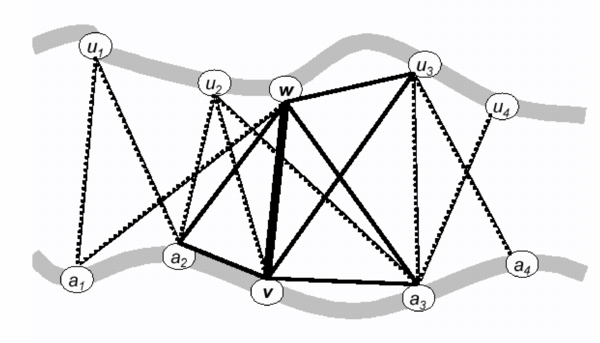
**The GARP refinement procedure**. Schematic example for the calculation of a mutual clustering coefficient (*C*_*vw*_) and window averaging. Residues (nodes) are shown as circles, predicted interactions (edges) are shown as black lines, and part of the protein backbone sequence is shown as thick grey lines. The analyzed edge (*v*, *w*) is shown in bold with some other edges of the graph. Edges, which connect nodes *v *and *w *through a third node, are shown as regular lines. The mutual clustering coefficient compares the number of these edges, or their integrated weights, with the number, or weights, expected from all edges, in which *v *or *w *are present. The five top and five bottom nodes form the sequence window for nodes *w *or *v*, respectively. Edge (*v*, *w*) is given the mean *C*_*vw *_value of all edges between the two windows.

To seek edges with high neighbourhood cohesiveness (*i.e*., that are part of a well connected graph regions) we used mutual clustering coefficient measures (*C*_*vw*_). For each edge between nodes *v *and *w*, *C*_*vw *_compares the number of edges that connect nodes *v *and *w *through one additional node with the number of such connecting edges expected from all the edges, in which *v *and *w *participate [13]. The *C*_*vw *_measures described by Goldberg and Roth are for unweighted edges and differ by the calculation of the expected number of edges. We introduce the *C*_*vw *_measure that uses edge weights, as detailed in the Methods section. To identify edges between sequence regions that are well connected, we define a sequence window centred on each node, and give each edge the mean of all the *C*_*vw *_scores of the edges between positions in the windows of its two nodes (Figure 1).

Our Graph Analysis Refinement of Protein-contacts (GARP) approach was examined with the Jaccard, and Geometric *C*_*vw *_measures for unweighted graphs [14] (formula (1), Methods), and, with a weighted Jaccard *C*_*vw *_measure (formula (2), Methods). This last measure was calculated as the difference between the weights' sum of the edges connecting nodes *v *and *w *through any third node, and the weights' sum of all edges with nodes *v *or *w *(excluding edge (*v*, *w*) itself). A difference was used instead of a ratio since the edge weights we use can be log-odds ratios [3].

We also examined the number of top scoring prediction used to create the graph. It can be defined by a threshold score, as a fraction of the number of all possible predictions, or as a fraction of the protein/MSA length (*L*). Finally, the width of the sequence window to average the *C*_*vw *_values was examined using a window size (*W*), which is the number of residues on each side of a node (with nodes at the sequence ends having windows shorter then 2*W*+1).

We tested GARP on a basic intra-protein contact prediction method we recently developed, P2PConPred [3]. Only sequence positions separated by at least six amino acids were considered. To optimize the GARP procedure we analyzed the P2PConPred predictions on a training set of 59 MSAs [3]. We found a high correlation between the Jaccard, Meet_Min and Geometric unweighted *C*_*vw*_. Such similarity between the performances of these measures was previously observed [13]. We thus further used only the Geometric unweighted and Jaccard weighted *C*_*vw *_measures. Graph edge selection was examined by using different fractions of the top prediction scores (0.25, 0.20, 0.15, 0.10, 0.05 or 0.01), or by taking predictions with scores equal or above a given z-score (1.0, 1.5, 2.0, 2.5, 3.0, 3.5 or 4). Tested window sizes were five or seven residues (*W *= 2 or *W *= 3, respectively), which are shorter than typical helices and strands. Evaluations were done with the top scoring *L*/10 pairs as usually done in other contact prediction studies [2-6]. We examined the results for all protein positions, and for MSA positions predicted to be in the protein core.

Optimal parameters for the training set were found to be: the 5% top basic scores, a window of five residues (*W *= 2), and applying the Geometric unweighted *C*_*vw*_. This combination gave a mean accuracy of 14% for all the protein, and 24% in the predicted core region. This improves the accuracy of P2PConPred for the whole protein and for core regions (Table 1 and additional file 1).

An independent test set was used to evaluate our GARP procedure using the above parameters with input from the P2PConPred (Table 2 and additional file 2). Accuracies significantly improved by 1.5 times, to 18% for the entire protein and by 1.08 times to 26% for predicted core regions using GARP. Finally, the results on the test set were compared with the results of the PoCM method of Hamilton *et al*. which integrates basic contact predictions using a neural network [6] (Table 2). PoCM is more accurate then GARP on whole proteins (23% vs. 18%), but is less accurate then GARP (and other measures) for core regions (16% vs. 26%).

## Discussion

The GARP procedure notably improved the accuracy of a basic intra-protein contacts prediction method. Our approach treats the basic predictions as weighted edges to construct an undirected graph. This allows the use of various graph analysis measures and facilitates further analyses (such as window averaging). As such, the approach is easy to implement and to test diverse measures that can further refine the accuracy of protein contact prediction.

The optimal parameters found for the procedure were based on a large training set. The optimal window size is the same as that found for the PoCM method [6], and the Geometric unweighted *C*_*vw*_, found optimal, is related to a metric used in 'signature algorithm' devised to identify transcription modules [14]. Using the top 5% basic scores to create the analyzed graph seems to balance the ratio between the retained true to false positive basic predictions. The *C*_*vw *_measure and its window averaging, then extract the likely true predictions from the graph. We note that the top scores threshold we used, was sufficient to generate a topologically informative graph, since the Geometric *C*_*vw *_measure does not use the graph edge weights.

The procedure is demonstrated here to improve predictions of P2PConPred, but it could easily be applied to other methods with little conceptual or technical limitations. Output of different present and future basic methods for contact prediction could be used as input for the graph construction.

The small improvement in accuracy for core regions might be related to the smaller number of edges possible within the predicted cores. Furthermore, the core prediction accuracy is initially high (~22–24%), challenging further improvements. However, even an improvement of one or two percent in this zone can have major effects on the modelling of protein structures using their predicted intra-protein contacts [15].

We found the PoCM method more accurate for entire protein than for core regions. This could reflect the presence of many more highly conserved positions in the core, and their limited prediction usefulness for that method. Nevertheless, PoCM performed very well on entire proteins, indicating a possible synergism between its approach and the one we described here.

## Methods

### Contact prediction methods

Our refinement procedure was tested on the P2PConPred [3] contact prediction method. Both methods score the contact likelihood for pairs of protein positions. P2PconPred was used as described in [3] with a pair-to-pair substitution matrix derived from the Blocks database release 13 [16]. Predictions were taken for positions at least six amino acids apart on the sequence.

### Predicted solvent accessibility

Core residues were predicted by the SABLE method [12] as previously described by Eyal *et al*. [3]. Core regions were defined as the set of all residues with predicted relative solvent accessibility smaller then 0.15.

### Mutual clustering coefficient

Edges in highly connected graph regions were identified by the following mutual clustering coefficients (*C*_*vw*_) described by Goldberg and Roth [13]:

Jaccard Index : *C*_*vw *_= |*N*(*v*) ∩ *N*(*w*)|/|*N*(*v*) ∪ *N*(*w*)|.

MeetMin : *C*_*vw *_= |*N*(*v*) ∩ *N*(*w*)|/min(|*N*(*v*)|, |*N*(*w*)|).     (1)

Geometric: *C*_*vw *_= |*N*(*v*) ∩ *N*(*w*)|^2^/|*N*(*v*)|·|*N*(*w*)|.

with *N*(*v*), the neighbours of node *v *in graph *G*, is defined as: *N*(*v*) = {*u *| *uv *∈ *G*}.

We introduce an additional mutual correlation coefficient *C*_*vw*_, called Jaccard weighted for use on weighted graphs:

Cvw=∑u∈N(v)∩N(w)(wgt(u,v)+wgt(u,w))−(∑u∈N(v),u≠wwgt(u,v)+∑u∈N(w),u≠vwgt(u,w))     (2)
 MathType@MTEF@5@5@+=feaafiart1ev1aaatCvAUfKttLearuWrP9MDH5MBPbIqV92AaeXatLxBI9gBaebbnrfifHhDYfgasaacH8akY=wiFfYdH8Gipec8Eeeu0xXdbba9frFj0=OqFfea0dXdd9vqai=hGuQ8kuc9pgc9s8qqaq=dirpe0xb9q8qiLsFr0=vr0=vr0dc8meaabaqaciaacaGaaeqabaqabeGadaaakeaacqWGdbWqdaWgaaWcbaGaemODayNaem4DaChabeaakiabg2da9maaqafabaGaeiikaGIaem4DaCNaem4zaCMaemiDaqNaeiikaGIaemyDauNaeiilaWIaemODayNaeiykaKIaey4kaSIaem4DaCNaem4zaCMaemiDaqNaeiikaGIaemyDauNaeiilaWIaem4DaCNaeiykaKIaeiykaKcaleaacqWG1bqDcqGHiiIZcqWGobGtcqGGOaakcqWG2bGDcqGGPaqkcqGHPiYXcqWGobGtcqGGOaakcqWG3bWDcqGGPaqkaeqaniabggHiLdGccqGHsislcqGGOaakdaaeqbqaaiabdEha3jabdEgaNjabdsha0jabcIcaOiabdwha1jabcYcaSiabdAha2jabcMcaPiabgUcaRmaaqafabaGaem4DaCNaem4zaCMaemiDaqNaeiikaGIaemyDauNaeiilaWIaem4DaCNaeiykaKcaleaacqWG1bqDcqGHiiIZcqWGobGtcqGGOaakcqWG3bWDcqGGPaqkcqGGSaalcqWG1bqDcqGHGjsUcqWG2bGDaeqaniabggHiLdGccqGGPaqkaSqaaiabdwha1jabgIGiolabd6eaojabcIcaOiabdAha2jabcMcaPiabcYcaSiabdwha1jabgcMi5kabdEha3bqab0GaeyyeIuoakiaaxMaacaWLjaWaaeWaaeaacqaIYaGmaiaawIcacaGLPaaaaaa@8FFD@

where *wgt*(*v*, *w*) is a weight (contact log-likelihood score) of the edge. Note that edge (*v*, *w*) is not a part of either term.

### Data sets

A training set of 59 protein families was taken for a list of known protein monomers [17]. Multiple sequence alignments (MSA) for these proteins were taken from the Pfam database [18]. MSAs with less than 15 sequences, more than 50% gaps and very short alignments of less than 25 residues were excluded. Our test set was taken from the work of Vicatos et al. and included 57 proteins from all SCOP classes [19]. The two sets were found dissimilar to each other by comparing their MSAs with the COMPASS profile-to-profile alignment method [20] using a threshold of 10^-3^.

### Calculation of z-score for the GARP edge selection

For each protein family from the training set, a mean and a standard deviation of the P2PConPred scores were calculated for all predicted contacts. Z-score of the edge was calculated as a number of standard deviation away the family mean. Graph edge selection was examined by taking predictions with scores equal or above a given z-score.

### GARP accuracy evaluation

GARP results were evaluated by accuracy (selectivity), which is the ratio between the number of true predicted contacts and the total number of predicted contacts.

## Competing interests

The authors declare that they have no competing interests.

## Authors' contributions

SP conceived and supervised the project. MFM and RM implemented the method, assembled the datasets, and tested the method. All authors analyzed the results and wrote the article.

## Supplementary Material

Additional File 1 GARP accuracies for P2PconPred on the training set. Click here for file

Additional File 2 GARP accuracies for orP2PconPred on the test set. Click here for file

## Figures and Tables

**Table 1:** L/10 best scores accuracy improvements of GARP contact prediction for P2PconPred on the training set using parameters: 5% top scores, *W *= 2, Geometric unweighted mutual clustering coefficient.

	**Without GARP procedure**		**With GARP procedure**	
	**Accuracy protein**	**Accuracy core**	**Accuracy protein**	**Accuracy core**

**P2PConPred**	0.12 ± 0.018	0.22 ± 0.024	0.14 ± 0.020	0.24 ± 0.029

**Table 2:** L/10 best scores accuracy improvements of GARP contact prediction for P2PconPred on the test set using parameters: 5% top scores ^a^, *W *= 2, Geometric unweighted mutual coefficient.

	**Without GARP procedure**		**With GARP procedure**	
	**Accuracy protein**	**Accuracy core**	**Accuracy protein**	**Accuracy core**

**P2PConPred**	0.12 ± 0.018	0.24 ± 0.027	0.18 ± 0.024	0.26 ± 0.030
**PoCM**	0.23 ± 0.027	0.16 ± 0.021	-	-

^a ^In cases where less then L/10 predictions where reported by GARP, the calculation was repeated starting with the top 10% of scores. This was necessary for no more then five families in predicting contacts within the core.
